# Are anxious and mixed depression two sides of the same coin? Similarities and differences in patients with bipolar I, II and unipolar disorders

**DOI:** 10.1192/j.eurpsy.2023.2445

**Published:** 2023-09-12

**Authors:** Antonio Tundo, Laura Musetti, Sophia Betrò, Erika Cambiali, Rocco de Filippis, Donatella Marazziti, Federico Mucci, Luca Proietti, Liliana Dell’Osso

**Affiliations:** 1 Istituto di Psicopatologia, Rome, Italy; 2 Department of Clinical and Experimental Medicine, University of Pisa, Pisa, Italy; 3 Department of Biotechnology, Chemistry and Pharmacy, University of Siena, Siena, Italy

**Keywords:** anxious depression, anxious distress specifier, major depressive episode, mixed depression

## Abstract

**Background:**

Diagnostic criteria are not always useful to discriminate major depression with anxious distress (ADS-D; Diagnostic and Statistical Manual for Mental Disorders, version-5 [DSM-5] criteria) from mixed depression (Koukopoulos’ criteria; KMX-D). So, clinicians need alternative tools to improve their diagnostic ability and to choose the most appropriate treatment. The aim of the present study is to identify socio-demographic and clinical features that discriminate patients with ADS-D from those with KMX-D.

**Methods:**

Two hundred and forty-one consecutive outpatients with unipolar (51%) and bipolar (49%) disorder, fulfilling DSM-5 criteria for a current major depressive episode (MDE) and with a 21-item Hamilton Depression Rating Scale score ≥ 14, were recruited and treated in a prospective observational study.

**Results:**

Ten percent of patients met criteria for KMX-D, 22% ADS-D, and 37% for both. Irritable premorbid temperament, mixed depression polarity at onset, mixed depression recurrence, and a high number of mania symptoms at intake were typical features of patients with KMX-D. Depressive polarity at onset, a low number of mania symptoms at intake, and generalized anxiety disorder comorbidity were typical features of patients with ADS-D. Multinomial logistic regression confirmed that higher rate of irritable temperament and higher Young Mania Rating Scale total score differentiated patients with KMX-D from patients with pure MDE.

**Conclusion:**

Our findings suggest some clinical features that could help differentiate between ADS-D and KMX-D in patients meeting both conditions and to select the appropriate treatment. However, the small sample size may have limited the power to detect differences between the groups. Further research is needed to confirm the results of present study.

## Introduction

In 2013, the Diagnostic and Statistical Manual for Mental Disorders, version-5 (DSM-5) [1] included the specifier “with anxious distress” for major depressive episode (MDE) in both major depressive disorder (MDD) and bipolar disorder (BD). This specifier encompasses the presence of at least two of the following symptoms during most of the days: (a) feeling keyed up or tense; (b) feeling unusually restless; (c) difficulty concentrating because of worry; (d) fear that something awful may happen; and (e) feeling that the individual might lose control of himself or herself. Several studies supported the validity of the DSM-5 criteria for MDE with anxious distress (ADS-D), and showed that it is a common clinical presentation with a prevalence ranging between 54 and 78% [2–7].

Compared with depression without anxious distress, ADS-D is characterized by higher rates of BD family history, hyperthymic temperament and of suicidal ideation, as well as a greater severity of the disease, higher number of hospitalizations, greater frequency of antidepressants (ADs) side effects and poor ADs response, and higher rates of chronicity [2–5, 7, 8]. Prevalence and clinical characteristics of ADS-D are quite similar to those of mixed depression, suggesting the presence of at least partial overlap between these sub-types of depression [9–11].

In a previous study, Tundo et al. [12] analyzed the relationship among ADS-D and mixed depression symptoms in patients with unipolar and bipolar I and II depression. To make a diagnosis of mixed depression, the authors initially employed DSM-5 criteria and Koukopoulos’ criteria [13] but the small number of patients meeting the DSM criteria (2.5%), consistent with previous studies (0–7.5%) [14–16], did not allow to use this subgroup for statistical analyses. Koukopoulos’s criteria, validated by Sani et al. [17] and extensively used in clinical practice since 1992 [18], consist in the presence of three or more of the following symptoms during a MDE (in MDD or BD): (a) psychic agitation or inner tension; (b) racing or crowded thoughts; (c) irritability or unprovoked feelings of rage; (d) absence of retardation; (e) talkativeness; (f) dramatic description of suffering or frequent spells of weeping; (g) mood lability and marked emotional reactivity; and (h) early insomnia. Tundo et al.’s study [12] confirmed that ADS-D and MDE with Koukopoulos’ criteria for mixed depression (KMX-D) are two overlapping conditions with 90 of 241 patients showing simultaneously both.

Therefore, the previous research confirmed the overlap but left one important question open: do ADS-D and KMX-D denote the same condition requiring the same treatment or two different conditions that diagnostic criteria (as they currently stand) are not always able to fully differentiate? Should this question be answered, the clinician would benefit from a more precise subtyping of depression.

The aim of this study was to identify the socio-demographic and clinical related features differentiating patients with ADS-D from those with KMX-D.

## Methods

### Subjects

This observational study included a cohort of 241 patients consecutively recruited from January 2015 to January 2016 at the Section of Psychiatry, Department of Clinical and Experimental Medicine, University of Pisa, Italy and at the Institute of Psychopathology in Rome, Italy, two Italian centers specialized in mood and anxiety disorders. Inclusion criteria were: (a) age 18–75 years; (b) meeting DSM-5 diagnostic criteria for lifetime MDD, single or recurrent episode, or for bipolar I (BD-I) or II (BD-II) disorder [1]; (c) fulfilling DSM-5 criteria for a current MDE [1]; and (d) a 21-item Hamilton Depression Rating Scale (HDRS_21_) [19] total score ≥ 14 at intake. Only the first observed MDE (index depressive episode) was considered for patients experiencing more than one MDE during the observational period. Exclusion criteria were substance/medication or medical/neurological induced mood disorders. Written informed consent for the anonymous use of clinical records was routinely collected at patients’ first visit. The procedure was approved by the local ethical committee and is in accordance with the Helsinki declaration of 1975 as revised in 2008.

### Assessments

All subjects underwent initial diagnostic assessments using the Structured Clinical Interview for DSM 5 (SCID-5) [20]. The semi-structured interview for mood disorders (SIMD) [21] was used to collect participants’ socio-demographic and clinical data. SIMD was developed to collect in a structured way information on family history, age and polarity at onset, illness duration, previous number and polarity of episodes, suicide attempts in the current or previous episodes, psychotic symptoms, hospitalizations, manic/hypomanic switch, alcohol and/or substance use. Whenever possible, secondary clinical data, obtained from other informants or from medical records, were used to support patients’ information. Depressive symptoms were evaluated using the HDRS_21_; suicidality with the item 3 of HDRS_21_ (score ≤ 1 absent, score ≥ 2 present); (hypo)manic symptoms with Young Mania Rating Scale (YMRS) [22]; clinical status with Clinical Global Impression of Severity (CGI-s) and of Improvement (CGI-i) scales [23]; the overall level of functioning with Global Assessment of Functioning (GAF) [24]; the presence of MDE specifiers with DSM-5 criteria [1], the presence of mixed depression with Koukopoulos criteria validated by Sani et al. [17]. Treatment adherence was collected at each follow-up visit from patients’ and relatives’ report and coded as 1 if the patient had been taking at least 90% of the prescribed drugs between visits and 0 elsewhere. Overall adherence at each follow-up was computed the ratio between the number of adherent patients and the number of patients seen at that specific follow-up, multiplied by 100.

Temperament was assessed using the brief version of Temperament Evaluation of Memphis, Pisa, Paris, and San Diego (TEMPS-M) [25]. This self-report questionnaire includes 35 items rated on a Likert scale ranging from 1 to 5 (1 = not at all, 2 = a little; 3 = moderately; 4 = much, and 5 = very much) that evaluate affective temperaments, including predominantly depressive, cyclothymic, irritable, anxious, and hyperthymic subtypes. The rating scales were administered by SB, EC, RdF, and LP, four psychiatrists experienced in mood disorders not involved in the treatment and experienced in mood disorders. All patients underwent clinical assessments at intake (T0) and after 4 (T1), 8 (T2), and 12 (T3) weeks.

We defined remission as a HDRS_21_ total score < 7 after 12 weeks of treatment maintained for further 4 weeks, response as a ≥50% reduction of baseline HDRS_21_ total score at T3 maintained for further 4 weeks, improvement as a CGI-i score ≥ 2 (“much” or “very much improved”) at T3 maintained for almost 4 weeks. The choice to use sustained remission, response and improvement is in line with the recommendations of ISBD Task Force report on the nomenclature of course and outcome in BD [26].

For the purpose of the present study, we split patients into four groups: patients who met criteria for ADS-D (ADS-D), for KMX-D (KMX-D), for both (ADS-D + KMX-D), and for neither (pure-MDE).

### Treatments

In this observational study, the two senior authors (L.M. and A.T.) chose the pharmacologic intervention according to their own clinical experience and the international guidelines for the treatment of unipolar [27, 28] and bipolar depression [29, 30] at the time of patient’s enrollment. As usual in an observational setting, the treatment was personalized considering not only the nosological diagnosis (BD or MDD), but also the premorbid temperament, the previous course and treatments’ response, the specifiers of the index episode, age, sex and the medical and psychiatric comorbidity. Generally, mild–moderate unipolar depression was treated with a selective serotonin reuptake inhibitor (SSRI) AD and severe unipolar depression with a serotonin–norepinephrine reuptake inhibitor (SNRI) or tri-tetracyclic antidepressant (TCA). Bipolar depression was treated with a mood stabilizer (MS), mostly lithium, and/or a second-generation antipsychotic (SGA), mostly quetiapine, in patients meeting DSM-5 diagnostic criteria for rapid-cycling BD [1] or with a history of past (hypo)manic or mixed episodes emerging within 8 weeks after introducing an AD [or “treatment-emergent switch” according ISBD nomenclature [26]]. In patients with bipolar depression without rapid cycling course or past AD-induced switch, ADs (SSRI) were used in combination with a MS and/or an SGA, prescribing SNRI or TCA as second choice [29, 31]. In patients with mixed depression MS (mostly valproate or carbamazepine) and/or SGA (mostly quetiapine) were used. Augmentation with AD (mostly SSRI) was used only if the depressive symptoms did not recover. This prescribing pattern is in line with that suggested by Stahl et al. [11] for mixed depression. For resistant unipolar or bipolar depression, corresponding to at least the level III of Thase and Rush [32], we adopted combination or augmentation strategies [33].

### Statistical analysis

Demographic and clinical characteristics of patients with ADS-D, KMX-D, ADS-D + KMX-D, and pure-MDE were compared using χ^2^ test or Fisher’s exact test for categorical variables, ANOVA for continuous variables and Kruskal–Wallis test (K–W test) for continuous or ordinal variables with a skewed distribution. To characterize the differential profile of demographic and clinical characteristics of the clinical groups, we conducted a multinomial logistic regression using as independent variables the characteristics significantly different between subgroups in univariate analyses. In this analysis, pure depression was used as the reference group. Following significant tests, post hoc comparisons were performed with Benjamini–Hochberg adjusted probability levels. Statistical analyses were conducted using IBM SPSS Statistical Version 21. All tests were two-tailed and the significance levels were set to *p* < 0.05.

## Results

### Study sample

The study sample of 241 patients included 118 BD (49%), of whom 31 (13%) were BD-I and 87 (36%) BD-II, and 123 (51%) MDD, of whom 81 (34%) suffered from recurrent and 42 (17%) from single episodes. Patients were mostly women (*n* = 180, 75%), and married (*n* = 146, 61%). Half of patients were regularly employed (*n* = 117, 49%). The age (mean ± SD) was 47.7 ± 13.6 years, the educational level (mean ± SD) 13.4 ± 4.1 years, the length of the illness (mean ± SD) was 15.4 ± 12.7 years. Fifty-three patients (22%) met criteria for ADS-D, 24 (10%) for KMX-D, 90 (37%) for ADS-D + KMX-D, and 74 patients (31%) for pure-MDE.

### Demographic, clinical characteristics, and drug treatment of patients with KMX-D, ADS-D, ADS-D + KMX-D, and pure-MDE

As shown in Table 1, patients with KMX-D differed significantly from those with ADS-D in terms of marital status (less frequently married), premorbid temperament (more irritable), polarity of onset (more frequently mixed depression, less frequently pure depression), and higher mean YMRS total score at study entry. They significantly differed from those with ADS-D + KMX-D on premorbid temperament (more irritable) and polarity of onset (higher frequency of mixed depression), and from those with pure-MDE as regard premorbid temperament (more irritable), polarity of onset (higher frequency of mixed depression, lower frequency of pure depression), polarity of previous recurrences (higher frequency of mixed depression), and higher mean YMRS total score at study entry.

**Table 1.** Demographic and clinical characteristics of patients with anxious depression (ADS-D), mixed depression (KMX-D), anxious and mixed depression (ADS-D + KMX-D), and pure depression (pure-MDE)

Variable	ADS-D (*N* = 53)	KMX-D (*N* = 24)	ADS-D + KMX-D (*N* = 90)	Pure-MDE (*N* = 74)	χ^2^ or KW	*p*-Value	Post hoc significant comparisons
Sex, female, %	72.0	76.0	79.3	70.3	2.0	0.565	
Age, years, mean (SD)	51.4 ± 12.8	44.4 ± 12.7	47.3 (12.9)	46.9 ± 14.9	1.8	0.139	
Married/living with partner (%)	72.0	36.0	64.1	56.8	15.1	**0.019**	ADS-D > KMX-D
Years of education, mean (SD)	13.0 ± 4.7	14.1 ± 3.2	13.4 ± 4.1	13.3 ± 4.1	0.416	0.741	
Employed (%)	46.0	52.0	50.0	47.3	1.4	0.963	
Diagnosis (%)					16.1	0.064	
Bipolar I	6.0	12.0	16.3	13.5			
Bipolar II	42.0	28.0	43.5	25.7			
Major depressive disorder, recurrent	30.0	36.0	23.9	47.3			
Major depressive disorder, single episode	22.0	24.0	16.3	13.5			
Family history (%)					19.1	0.085	
Absent	22.0	20.0	28.3	12.2			
Depression	30.0	48.0	28.3	44.6			
Bipolar	36.0	20.0	34.8	23.0			
Anxiety	12.0	8.0	7.6	17.6			
Psychosis	0	4.0	1.1	2.7			
Temperaments, mean (SD)							
Dysthymic	0.61 ± 0.18	0.65 ± 0.22	0.58 ± 0.20	0.54 ± 0.18	2.3	0.075	
Cyclothymic	0.50 ± 0.23	0.57 ± 0.21	0.50 ± 0.22	0.50 ± 0.18	3.0	0.386	
Hyperthymic	0.52 ± 0.17	0.58 ± 0.24	0.59 ± 0.20	0.54 ± 0.18	1.7	0.166	
Irritable	0.36 ± 0.15	0.58 ± 0.20	0.44 ± 0.18	0.37 ± 0.14	25.9	**<0.001**	KMX-D > pure-MDE, ADS-D, ADS-D + KMX-D
Anxious	0.47 ± 0.17	0.50 ± 0.21	0.45 ± 0.18	0.43 ± 4.7	1.9	0.580	
Lifetime comorbidity (%)							
Obsessive compulsive disorder	8.0	24.0	17.4	17.6	3.8	0.284	
Panic disorder	24.0	20.0	27.2	29.7	1.1	0.773	
Social anxiety disorder	6.0	4.0	4.3	6.8	0.6	0.897	
Generalized anxiety disorder	10.0	0	5.4	0	8.9	**0.03**	ADS-D > pure-MDE
Eating disorders	12.0	8.0	17.4	10.8	2.4	0.490	
Somatoform disorders	4.0	0	2.2	2.7	1.2	0.763	
Alcohol abuse	14.0	24.0	18.5	13.5	2.0	0.577	
Substance abuse	12.0	24.0	15.2	16.2	1.8	0.606	
Cannabis	8.0	16.0	5.4	9.5	3.1	0.380	
Cocaine	2.0	12.0	3.3	8.1	5.6	0.161	
Heroin	0	0	0	0	na	na	
Benzodiazepine	4.0	4.0	8.7	2.7	3.3	0.347	
Age at onset, years, mean (SD)	35.9 ± 14.2	31.6 ± 13.3	31.6 ± 12.9	30.99 ± 14.0	3.9	0.268	
First episode polarity (%)					39.9	**<0.001**	
Pure depression	92.0	36.0	65.2	79.7			Pure-MDE > KMX-D; ADS-D > KMX-D; and ADS-D + KMX-D
(Hypo)mania	2.0	12.0	9.8	10.8			
Mixed mania	2.0	0	2.2	0			
Mixed depression	4.0	52.0	22.8	9.5			KMX-D > pure-MDE, ADS-D, ADS-D + KMX-D; ADS+MIX > ADS-D
Length of illness, years, mean (SD)	16.1 ± 14.5	12.9 ± 9.9	15.4 ± 12.7	15.72 ± 12.3	0.5	0.922	
Number of previous episodes, mean (SD)							
Pure depressive	3.9 (5.5)	2.6 (3.9)	2.8 (4.4)	3.40 (4.07)	4.3	0.228	
Manic	0.26 (1.4)	0.20 (0.65)	0.19 (0.85)	0.40 (1.53)	2.0	0.578	
Hypomanic	1.9 (3.4)	1.6 (3.3)	2.1 (3.7)	0.92 (2.14)	6.2	0.101	
Manic with mixed features	0.70 (4.2)	0.17 (0.48)	0.08 (0.31)	0.01 (0.12)	5.9	0.116	
Depressive with mixed features	0.58 (1.1)	2.8 (6.8)	1.3 (2.7)	1 (3.17)	12.6	**0.006**	KMX-D > pure-MDE
Total	6.6 (8.8)	7.0 (8.7)	6.6 (8.5)	5.60 (6.65)	0.3	0.968	
Suicide attempts, %	16.0	28.0	18.5	12.2	3.5	0.315	
Switch, %	16.0	12.0	21.7	13.5	4.3	0.633	
Lifetime delusions, %	18.0	16.0	25.2	14.9	14.9	0.248	
Index episode							
Duration, weeks, mean (SD)	25 (33.2)	65.8 (94.1)	36.1 (88.0)	35 (90.5)	6.7	0.081	
(HDRS_21_, mean (SD)	19.9 (4.5)	19.1 (3.8)	20.1 (4.3)	18.4 (3.9)	8.7	**0.034**	ADS-D + KMX-D > pure MDE
YMRS, mean (SD)	0.96 (1.8)	3.5 (3.1)	2.8 (3.1)	0.57 (1.3)	55.3	**<0.001**	KMX-D > ADS-D, pure-MDE; ADS-D + KMX-D > ADS-D, pure MDE
CGI-s, mean (SD)	4.5 (0.65)	4.6 (0.65)	5.0 (2.4)	4.5 (1.7)	12.9	**0.005**	ADS-D + KMX-D > pure-MDE
GAF, mean (SD)	51.3 (7.3)	51.4 (4.5)	51.3 (6.5)	52.4 (4.8)	1.8	0.623	

Abbreviations: χ^2^, chi-square test; CGI-s, Clinical Global Impression of Severity; GAF, Global Assessment of Functioning; HDRS_21_, Hamilton Depression Rating Scale; KW, Kruskal–Wallis test; SD, standard deviation; YMRS, Young Mania Rating Scale.

Patients with ADS-D significantly differed from those with ADS-D + KMX-D on the polarity at onset (lower frequency mixed depression, higher frequency of pure depression) and a lower mean YMRS total score at study entry, and from that with pure-MDE on the higher frequency of generalized anxiety disorder (GAD) comorbidity.

Lastly, patients with ADS-D + KMX-D had higher HDRS_21_ and YMRS total scores and CGI-s score at study entry than those with pure-MDE.

The multinomial logistic regression was conducted using as independent variables marital status, irritable temperament, comorbid GAD, number of previous depressive episodes with mixed features, HDRS_21_, YMRS, and CGI-s scores (the characteristics significantly different between subgroups in univariate analyses), and using pure-MDE as the reference. GAD comorbidity was not included because it yielded convergence problems. The result indicates that, compared with the reference group of pure-MDE, patients with KMX-D exhibited higher rates of irritable temperament and higher YMRS total scores and patients with ADS-D + KMX-D only higher YMRS total scores (Table 2).

**Table 2.** Multinomial logistic regression including characteristics significantly different between subgroups in univariate analysis^a^

Group		*b*	SE(*b*)	Wald	df	*p*	OR	95% confidence interval for OR	
								Lower bound	Upper bound
ADS-D	Intercept	−0.864	1.416	0.372	1	0.542			
	Irritability score	−0.005	0.041	0.013	1	0.910	0.995	0.918	1.079
	Number of depressive episodes with mixed features	−0.112	0.117	0.918	1	0.338	0.894	0.711	1.124
	HDRS_21_	0.102	0.054	3.523	1	0.061	1.107	0.996	1.231
	YMRS	0.208	0.139	2.245	1	0.134	1.232	0.938	1.617
	CGI severity	−0.281	0.280	1.005	1	0.316	0.755	0.436	1.308
	Unmarried	−0.656	0.423	2.411	1	0.120	0.519	0.227	1.188
	Married	0^b^			0				
KMX-D	Intercept	−3.977	2.054	3.750	1	0.053			
	Irritability score	0.186	0.050	13.928	1	**<0.001**	**1.204**	**1.092**	**1.327**
	Number of depressive episodes with mixed features	0.112	0.070	2.570	1	0.109	1.119	0.975	1.284
	HDRS_21_	0.036	0.076	0.219	1	0.639	1.036	0.893	1.203
	YMRS	0.527	0.139	14.434	1	**<0.001**	**1.693**	**1.290**	**2.222**
	CGI severity	−0.472	0.455	1.075	1	0.300	0.624	0.255	1.522
	Unmarried	0.801	0.568	1.984	1	0.159	2.227	0.731	6.785
	Married	0^b^			0				
KMX-D + ADS-D	Intercept	−3.183	1.129	7.956	1	0.005			
	Irritability score	0.064	0.037	3.047	1	0.081	1.066	0.992	1.146
	Number of depressive episodes with mixed features	0.052	0.064	0.663	1	0.416	1.053	0.930	1.193
	HDRS_21_	0.083	0.049	2.824	1	0.093	1.087	0.986	1.197
	YMRS	0.528	0.120	19.244	1	**<0.001**	**1.696**	**1.339**	**2.147**
	CGI severity	0.037	0.110	0.112	1	0.738	1.037	0.837	1.286
	Unmarried	−0.402	0.388	1.073	1	0.300	0.669	0.313	1.431
	Married	0^b^			0				

a The reference category is pure-MDE.

b This parameter was set to 0 because it is redundant.

Abbreviations: ADS-D, MDE with anxious distress; CGI severity, Clinical Global Impression of Severity; HDRS_21_, Hamilton Depression Rating Scale; KMX-D, mixed depression according Koukopoulos’ criteria; KMX-D + ADS-D, criteria for KMX-D and ADS-D; MDE, major depressive episode; YMRS, Young Mania Rating Scale.

Table 3 reports the baseline treatment. As expected, patients with KMX-D were prescribed less frequently than those with ADS-D and pure-MDE all classes of AD and more frequently MS. Moreover, compared to patients with pure-MDE, those with ADS-D + KMX-D were prescribed less frequently TCA and more frequently SGA and MS.

**Table 3.** Baseline treatment in patients with anxious depression (ADS-D), mixed depression (KMX-D), anxious and mixed depression (ADS-D + KMX-D), and pure depression (pure-MDE)

Drug	ADS-D (*N* = 53)	KMX-D (*N* = 24)	ADS-D + KMX-D (*N* = 90)	Pure-MDE (*N* = 74)
Antidepressants, %	93.9	56.0	78.3	86.5
SSRI, %	68.0	32.0	51.1	40.5
SSRI dosage^a^, mean (SD)	31.0 (15.2)	35.0 (15.1)	29.4 (16.5)	36 (15.7)
TCA, %	30.0	24.0	21.7	41.9
TCA dosage, mean (SD)	64.1 (57.4)	100.8 (39.2)	79.3 (50.9)	97.2 (46.9)
SNRI, %	14.0	4.0	9.8	13.5
Dose SNRI, mean (SD)	186.4 (122.1)	0	119.2 (52.0)	133.5 (62.1)
Mirtazapine, %	8.0	0	4.3	2.7
Mirtazapine dosage, mean (SD)	15 (56.25)	0	15 (7.50)	0
Bupropion, %	2.0	0	1.1	2.7
Bupropion dosage, mean (SD)	150 (150–150)	0	300 (300–300)	225 (150–300)
Others antidepressant, %	4	0	4.3	5.4
Combination %	42.0	16.0	33.7	32.4
Combination (2 or more AD), %	34.0	4.0	18.5	23.0
Augmentation antidepressant + pramipexole, %	2.0	4.0	0	4.1
Pramipexole dosage, mean (SD)	0.7 (0.7–0.7)	0.7 (0.7–0.7)	0	0.72 (0.54–1)
Augmentation antidepressant + aripiprazole, %	8.0	8.0	7.6	10.8
Aripiprazole dosage, mean (SD)	3.8 (1.4)	3.8 (1.8)	5.4 (2.7)	3.9 (2.8)
Mood stabilizer, %	46	88	68.5	47.3
Lithium, %	24.0	16.0	28.3	20.3
Lithium serum level (mEq/l), mean (SD)	0.63 (0.18)	0.42 (0.06)	0.50 (0.21)	0.50 (0.2)
Valproic acid, %	26.0	44.0	37.0	23.0
Valproic acid dosage, mean (SD)	592.3 (418.8)	481.8 (202.8)	508.8 (208.7)	523.5 (244.4)
Carbamazepine, %	8.0	28.0	13.0	9.5
Carbamazepine dosage, mean (SD)	500.0 (270.8)	428.6 (213.8)	333.3 (65.1)	300 (115.5)
Lamotrigine, %	6.0	0	8.7	1.4
Lamotrigine dosage, mean (SD)	133.3 (38.2)	0	135.7 (55.6)	na
SGA, %	26.0	44.0	45.7	20.3
SGA dosage, mean (SD)	6.5 (5.0)	5.3 (4.0)	4.6 (4.6)	5.8 (4)
FGA, %	6.0	0	3.3	0
FGA dosage, mean (SD)	3.9 (3.5)	0	7.2 (4.9)	na

Abbreviations: FGA, first generation antipsychotic; SGA, second-generation antipsychotic; SNRI, serotonin–noradrenalin reuptake inhibitor; SSRI, selective serotonin reuptake inhibitor; TCA, tricyclic antidepressant.

a Fluoxetine equivalent.

### Outcomes of KMX-D, ADS-D, ADS-D + KMX-D, and pure-MDE patients

As shown in Table 4, the four study groups (KMX-D, ADS-D, ADS-D + KMX-D, and pure-MDE) did not significantly differ in the drop-out rate during follow-up and in the treatment-induced switch, while they significantly differ on treatment adherence (pure-MDE lower than ADS-D + KMX-D). After 12 weeks of treatment, no significant differences between groups were found concerning HDRS_21_ total score, YMRS total score, and GAF total score. No suicide attempts were committed during the follow-up.

**Table 4.** Outcomes after 12 weeks of treatment in patients with anxious depression (ADS-D), mixed depression (KMX-D), anxious and mixed depression (ADS-D + KMX-D), and pure depression (pure-MDE)

Outcome	ADS-D (*N* = 53)	KMX-D (*N* = 24)	ADS-D + KMX-D (*N* = 90)	Pure-MDE (*N* = 74)	χ^2^ or ANOVA	*p*-Value	Post hoc significant comparisons
Drop-out, %	42.0%	52.0%	37.0%	40.5%	1.8	0.595	
Induced switch, %	6.9	0.3	4.0	4.5	3.5	3.5	
Treatment adherence, %	100	91.7	100	88.6	9.9	**0.019**	ADS-D + KMX-D > pure-MDE
HDRS_21_ total score, Mean (SD)	7.2 (6.9)	7.1 (5.4)	9.1 (7.4)	8.1 (6.3)	1.3	0.739	
YMRS total score, mean (SD)	0.50 (1.2)	2.2 (4.7)	1.3 (2.3)	0.4 (1)	7.5	0.057	
GAF total score, mean (SD)	72.4 (10.9)	70.3 (13.3)	66.4 (15.6)	65.4 (12.5)	5.6	0.132	
Remission, %	65.5	50.0	44.8	54.5	3.4	0.328	
Response, %	75.9	50.0	36.0	61.4	13.9	**0.003**	ADS-D > ADS-D + KMX-D
Improvement, %	86.2	91.7	77.6	90.9	4.5	0.256	

Abbreviations: χ^2^, chi-square test; ANOVA, analysis of variance; GAF, Global Assessment of Functioning; HDRS_21_, Hamilton Depression Rating Scale; YMRS, Young Mania Rating Scale.

Patients with KMX-D, ADS-D, ADS-D + KMX-D, and pure-MDE depression did not significantly differ on remission rate and CGI improvement rate, whereas they significantly differ as regard the response rate (ADS-D higher than ADS-D + KMX-D).

## Discussion

The present study confirms that depression with anxiety features (DSM-5 criteria) and mixed depression (Koukopoulos’ criteria) are two very common MDE presentations, with a prevalence of 47% and 59%, respectively, – consistent with that reported in the literature [2–7, 9, 10] – that these features frequently overlap (39% of the sample) and that socio-demographic and clinical features may help to differentiate between them.

Specifically, patients with KMX-D are more likely to be single, to have a premorbid irritable temperament, mixed depression polarity at onset, previous mixed depression recurrence, and a higher number of mania symptoms (as measured by YMRS) at intake. On the contrary, being married, having a pure depressive polarity at onset, a low number of mania symptoms (as measured by YMRS) at intake and GAD comorbidity are more common among patients with ADS-D.

The higher YMRS total score for KMX-D patients was expected since five YMRS items are included in the KMX-D criteria (language-thought disorder, talkativeness, irritability, disruptive/aggressive behavior, and increased motor activity/restlessness). Similarly, the higher prevalence of GAD comorbidity in ADS-D patients was expected, since GAD includes all symptoms of ADS-D (excessive anxiety and worry, loss of control, restlessness or feeling keyed up or at the edge, difficulty concentrating because of worry).

The evidence of a high frequency of irritable temperament in patients with KMX-D is in contrast with the result of a previous study showing a relationship between KMX-D and hyperthymic temperament [34]. Differences in temperament assessment (using an ad hoc scale in our study, clinical criteria in Sani et al.’s study) and in the subtypes of depression compared (pure mixed depression, pure anxious depression, mixed and anxious depression and pure depression in our study, mixed and non-mixed depression in Sani et al.’s, study) could be the reason for this conflicting result.

Lastly, patients with KMX-D received more often MS and those with ADS-D all classes of AD or two ADs combination. At the end of follow-up (12 weeks) the two groups did not differ on outcomes and no suicide attempt was recorded.

Patients meeting both diagnoses, compared with those with pure-MDE, had more severe MDE, higher treatment adherence rate, received more frequently MS and SGA and less frequently TCA. The lower treatment adherence of patients with pure-MDE compared with patients with ADS-D + KMX-D could be explained by the difference in episode severity between the two groups. In fact, evidence from two studies [35, 36] suggests that a lower severity of depression predicts lower treatment adherence.

In conclusion, about one-half of patients with MDE meet DSM-5 criteria for depression with anxiety features or Koukopoulos’ criteria for mixed depression and many of these patients meet both diagnoses. Patients with both diagnoses are a heterogeneous group including persons with ADS-D and persons with KMX-D that the current diagnostic criteria are not able to fully differentiate.

A previous study, based on a network analysis, showed as possible cause of misdiagnosis that the KMX-D and ADS-D criteria are connected by two bridge symptoms, the first KMX-D criterion (*psychic agitation or inner tension*) and the first ADS-D criterion (*feeling keyed up or on edge*), which characterize two different psychopathological conditions [12]. The first KMX-D criterion describes a primary physical manifestation that secondarily makes patient very anxious and fearful [15], the first ADS-D criterion, derived from the similar item of GAD, indicates physical symptoms accompanying, or secondary to the excessive anxious expectation regarding routine life circumstances [1]. The subtle distinction between these two symptoms requires major semiological competences and is very hard to capture for a clinician, when dealing with patients presenting with a severe depressive episode. An additional difficulty to differentiate KMX-D from ADS-D could be that anxiety symptoms (apprehension, fear, preoccupation, somatic inner restlessness, and somatic anxiety) are frequent in mixed depression, mostly in unipolar mixed depression, and, although they are not the core features of this condition, they are related to manic features and to the severity of the episode [37].

The results of the present study, needing further confirmations, indicate some clinical features they may help in the differential diagnosis and treatment selection. Patients with KMX-D typically show irritable premorbid temperament, mixed depression polarity at onset, prevalence of mixed depression recurrences, high number of mania symptoms at intake. They respond preferentially to MS. On the contrary, depressive polarity at onset, low number of mania symptoms at intake, GAD comorbidity are typical features of patients with ADS-D. They respond preferentially to ADs.

The fuzzy boundary between KMX-D and ADS-D has important diagnostic and therapeutic implications. Diagnostic criteria, mostly the first KMX-D and first ADS-D items that, as currently stand, generate overlapping should be reconsidered and new diagnostic criteria should be validated.

Our results should be interpreted keeping in mind that the small sample size may have limited the power to detect differences among the 4 groups.

Moreover, further research is needed to identify clinical characteristics of patients with KMX-D and ADS-D who respond to ADs (indicative of anxious depression) and to MS and/or SGA (indicative of mixed depression).
